# Higher NICU admissions in infants born at ≥35 weeks gestational age during the COVID-19 pandemic

**DOI:** 10.3389/fped.2023.1206036

**Published:** 2023-07-07

**Authors:** Priya Jegatheesan, Sudha Rani Narasimhan, Angela Huang, Matthew Nudelman, Dongli Song

**Affiliations:** ^1^ Department of Pediatrics, Division of Neonatology, Santa Clara Valley Medical Center, San Jose, CA, United States; ^2^ Department of Pediatrics, Stanford University School of Medicine, Stanford, CA, United States; ^3^ Neonatology/Pediatrics, Mountain Health Network, Marshall University, Huntington, WV, United States

**Keywords:** COVID-19 pandemic, neonatal intensive care unit (NICU) admission, maternal hypertension, metabolic acidosis, exclusive breastfeeding

## Abstract

**Background:**

Increasing evidence has shown that the COVID-19 pandemic has had a profound negative impact on vulnerable populations and a significant effect on maternal and neonatal health. We observed an increase in the percentage of infants admitted to NICU from 8% to 10% in the first year of the pandemic. This study aimed to compare the delivery room outcomes, NICU admissions and interventions, and neonatal outcomes two years before and during the pandemic.

**Methods:**

This was a retrospective study in a public hospital between pre-COVID-19 (April 2018–December 2019) and COVID-19 (April 2020–December 2021). Data were obtained from all live births at ≥35 weeks gestation (GA). Maternal and neonatal demographics, delivery room (DR), and NICU neonatal outcomes were compared between the study periods using simple bivariable generalized estimating equations (GEE) regression. Multivariable GEE logistic regression analysis was performed to adjust for the effects of baseline differences in demographics on the outcomes.

**Results:**

A total of 9,632 infants were born ≥35 weeks gestation during the study period (pre-COVID-19 *n* = 4,967, COVID-19 *n* = 4,665). During the COVID-19 period, there was a small but significant decrease in birth weight (33 g); increases in maternal diabetes (3.3%), hypertension (4.1%), and Hispanic ethnicity (4.7%). There was a decrease in infants who received three minutes (78.1% vs. 70.3%, *p* < 0.001) of delayed cord clamping and increases in the exclusive breastfeeding rate (65.9% vs. 70.1%, *p* < 0.001), metabolic acidosis (0.7% vs. 1.2%, *p* = 0.02), NICU admission (5.1% vs. 6.4%, *p* = 0.009), antibiotic (0.7% vs. 1.7%, *p* < 0.001), and nasal CPAP (1.2% vs. 1.8%, *p* = 0.02) use. NICU admissions and nasal CPAP were not significantly increased after adjusting for GA, maternal diabetes, and hypertension; however, other differences remained significant. Maternal hypertension was an independent risk factor for all these outcomes.

**Conclusion:**

During the COVID-19 pandemic period, we observed a significant increase in maternal morbidities, exclusive breastfeeding, and NICU admissions in infants born at ≥35 weeks gestation. The increase in NICU admission during the COVID-19 pandemic was explained by maternal hypertension, but other adverse neonatal outcomes were only partly explained by maternal hypertension. Socio-economic factors and other social determinants of health need to be further explored to understand the full impact on neonatal outcomes.

## Introduction

The COVID-19 pandemic due to SARS-CoV-2 infection has had profound negative impacts on vulnerable populations (1). The effect of the pandemic on population health has been more than the direct effect of the SARS-CoV-2 infection itself. The pandemic has indirectly impacted the socio-economic status of the population, limited access to healthcare systems, and worsened social determinants of health (2–4). There is a significant effect on the overall health of pregnant mothers and newborns (5).

Our institution has had one of the lowest NICU admission rates, at around 8%, in the state of California for the last decade. However, we observed an increase in the percentage of infants being admitted to the neonatal intensive care unit (NICU) in the first year of the pandemic to 10%. The increase in NICU admissions in 2020 led us to evaluate the impact of the COVID-19 pandemic on the outcomes of all the deliveries in our institution. Infants born at <35 weeks GA are admitted to NICU for prematurity according to our admission policy. We have previously shown that in our public safety net hospital, there was no change in our center's very preterm birth rate or very low birth weight in 2020, the first year of the pandemic (6). We focused on the delivery room (DR) and neonatal outcomes of infants born ≥35 weeks GA to understand the impact of the pandemic on term and late preterm infants.

The objective of this study was to compare the DR outcomes, NICU admissions and interventions, and neonatal outcomes in infants born two years before and during the COVID-19 pandemic and evaluate the effect of any changes in maternal morbidities on these outcomes.

## Methods

This was a retrospective study comparing the DR and neonatal outcomes of all live births in a public hospital between the pre-COVID-19 period: 1 April, 2018–31 December 2019, and the COVID-19 pandemic period: 1 April 2020–31 December 2021. The rate of prematurity <37 weeks and <35 weeks gestational age (GA), and DR and NICU outcomes for infants born at ≥35 weeks GA were evaluated in detail to assess the impact of the COVID-19 pandemic on these infants. The study was approved by the Institutional Review Board.

### Newborn care practices

Infants born at <35 weeks GA or with birth weight <1,800 grams were routinely admitted to NICU for neonatal care. In the DR, the goal was to clamp the umbilical cord after three minutes to optimize neonatal transition and placental transfusion. The Umbilical cord was clamped earlier than three minutes if the infant was not breathing by one minute despite the initial steps of newborn care or if there was maternal hemorrhage. All stable infants ≥35 weeks GA were placed on mothers' chest for skin-to-skin in the DR. Skin-to-skin care was uninterrupted for the first hour to promote bonding and breastfeeding. Newborns roomed-in with the mothers in the postpartum unit at all times. Infants who required respiratory support, intravenous fluids, antibiotics, or those who had severe metabolic acidosis, abnormal neurological examination, or major anomalies were admitted to NICU for further evaluation and treatment.

### COVID-19 pandemic changes

Our institution implemented universal SARS-CoV-2 screening of all pregnant women who were admitted for delivery in April 2020 (7). From October 2020, women who tested positive within 90 days prior to admission for delivery and did not have new symptoms of COVID were not retested at the time of delivery. If the mother's infection was within 10–14 days of delivery, the mother and infant roomed in together with airborne isolation precautions, with the mother wearing a surgical mask when holding and breastfeeding the baby during the isolation period. Skin-to-skin and breastfeeding were encouraged. The infants born to mothers who were in the intensive care unit or too sick to care for their infant were separated from the mother and cared for by another healthy family member. There was no change in the NICU admission criteria or other NICU care practices during the pandemic.

The hospital visitation policy was restricted to one support person for the mother during the delivery process and in the postpartum unit. The NICU visitation policy was also restricted to only one designated parent at the beginning of the pandemic and six months later expanded to include both parents but only one parent at the bedside at a time. Parents were not allowed to visit NICU during the SARS-CoV-2 isolation period if they were positive or during the quarantine period if they were exposed. The lactation consultant coordinated the delivery of the mother's own milk when the parents were in isolation or quarantine. The parents were able to see their infant via web camera and communicate with the NICU staff via phone.

### Data collection

Data were obtained from automated reports and chart reviews for all births from hospital electronic health records. Data for all NICU admissions were obtained from the NICU database that is maintained for mandated NICU data submissions and quality improvement projects. The maternal demographics included age, gravida, para, maternal diabetes, hypertension, pre-pregnancy BMI, race and ethnicity, SARS-CoV-2 infection, chorioamnionitis; DR outcomes included delivery type, cord blood gas values, duration of delayed cord clamping (DCC), APGAR scores, DR intubation, and chest compressions; neonatal demographics included GA, birth weight, and sex; and neonatal outcomes included any breastfeeding, exclusive breastfeeding, NICU admissions, reasons for NICU admission, and NICU interventions including antibiotics use, respiratory support including mechanical ventilation, continuous positive airway pressure (CPAP), non-invasive mechanical ventilation (NIMV), inhaled nitric oxide (iNO), and blood transfusions.

### Analysis

Maternal and neonatal demographics, DR, and NICU neonatal outcomes were compared between the study periods using simple bivariable generalized estimating equations (GEE) regression models clustered around unique pregnancies to account for multiples (i.e., twins, triplets, etc.). Gaussian, Poisson, and logistic GEE models were used for continuous, count, and binary outcomes, respectively. Multivariable GEE logistic regression analysis was performed to adjust for the effects of baseline differences in demographics on the outcomes. All GEE models utilized independent within-group correlation structures and robust variance estimates. Data analysis was performed using Stata 17.0 (Stata Corp, College Station, TX) and *p*-value <0.05 was considered significant.

## Results

A total of 9,632 were born at ≥35 weeks GA (pre-COVID-19 *n* = 4,967, COVID-19 *n* = 4,665) and included in the study. There was no difference in the rate of prematurity <35 weeks GA (3.5% vs. 4.0%, *p* = 0.2) between the two study periods. The demographics and DR outcomes data are shown in Table 1. During the COVID-19 period, there was a small but statistically significant decrease in birth weight (33 g) of infants born at ≥35 weeks gestation. There was no difference in maternal age, gravida, or parity between the study periods, but there were significant increases in maternal diabetes (21.2% vs. 24.5%), maternal hypertension (21.9% vs. 25.9%), and Hispanic ethnicity (68.4% vs. 73.1%) during the pandemic. During the pandemic, 4% of the mothers in this study were SARS-CoV-2 positive during pregnancy.

**Table 1:** Demographics.

	2018–2019	2020–2021	Difference (95% CI)	*p*-value
	(Pre-COVID-19)	(COVID-19)		
Liveborn infants ≥35 weeks GA, *n*	4,967	4,665		
Infant demographics				
Gestational age, weeks, mean (SD)	39.2 (1.3)	39.0 (1.2)	-0.2 (−0.3, −0.2)	<0.001
Birth weight, grams, mean (SD)	3,355 (489)	3,322 (482)	−33 (−53, −13)	0.001
Male sex, *n* (%)	2,524 (50.8)	2,382 (51.1)	0.2 (−1.8, 2.3)	0.8
Maternal demographics				
Age, mean (SD)	29.6 (6.2)	29.4 (6.3)	−0.1 (−0.4, 0.1)	0.3
Gravida, median (IQR)	2 (1, 4)	2 (1, 4)	0.0 (−0.1, 0.1)	0.9
Parity, median (IQR)	2 (1, 3)	2 (1, 3)	0.0 (0.0, 0.1)	0.2
Multifetal pregnancies, *n* (%)	106 (2.1)	104 (2.2)	0.1 (−0.7, 0.9)	0.8
Twins, *n* (%)	100 (2.0)	104 (2.2)		
Triplets, *n* (%)	6 (0.1)	0 (0)		
Diabetes, *n* (%)^a^	1,049 (21.2)	1,137 (24.5)	3.3 (1.6, 5.0)	<0.001
Gestational, *n* (%)	935 (18.9)	1,066 (23)		
Pre-gestational, *n* (%)	114 (2.3)	71 (1.5)		
Hypertension, *n* (%)^a^	1,089 (22.0)	1,210 (26.1)	4.1 (2.4, 5.9)	<0.001
Gestational, *n* (%)	413 (8.3)	517 (11.1)		
Chronic, *n* (%)	112 (2.3)	147 (3.2)		
Pre-eclampsia/Eclampsia, *n* (%)	564 (11.4)	546 (11.8)		
Body mass index, mean (SD)^b^	27.9 (6.7)	28.2 (6.6)	0.2 (0.0, 0.5)	0.09
Race and ethnicity				<0.001
Hispanic, *n* (%)	3,399 (68.4)	3,412 (73.1)		
Asian, *n* (%)	736 (14.8)	546 (11.7)		
White, *n* (%)	418 (8.4)	328 (6.9)		
Black, *n* (%)	248 (5.0)	199 (4.3)		
Multiracial, *n* (%)	92 (1.9)	91 (2.0)		
Pacific Islander, *n* (%)	37 (0.7)	31 (0.7)		
Unknown, *n* (%)	27 (0.5)	56 (1.2)		
Native American, *n* (%)	8 (0.2)	9 (0.2)		
Other, *n* (%)	2 (0.0)	1 (0.0)		
Chorioamnionitis, *n* (%)	405 (8.2)	392 (8.5)	0.3 (−0.8, 1.4)	0.6
Cesarean section, *n* (%)	1,332 (26.8)	1,249 (26.8)	0.0 (−1.9, 1.8)	1.0
SARS-CoV-2 infection, *n* (%)	0 (0)	186 (4)	n/a	n/a

^a^ A total of 10 patients from Pre-COVID-19 and 26 patients from COVID-19 period were excluded from difference analysis due to missing data.

^b^ A total of 187 patients from Pre-COVID-19 and 272 patients from COVID-19 period were excluded from difference analysis due to missing data.

### Delivery room outcomes

There was a decrease in infants who received at least one minute DCC (96.3% vs. 95.1%), and completed three minutes DCC (78.1% vs. 70.3%), but an increase in the severe metabolic acidosis (base deficit ≥16) (0.7% vs. 1.2%) in umbilical cord gas during the pandemic as shown in Table 2. The increase in severe metabolic acidosis remained significant even after adjusting for baseline differences in GA, maternal diabetes, and hypertension. There was no change in the APGAR scores, DR intubation, or cardiac medications between the study periods.

**Table 2:** Delivery room and neonatal outcomes.

	2018–2019	2020–2021	Difference (95% CI)	*p*-value
	(Pre-COVID-19)	(COVID-19)		
Livebirths ≥ 35 weeks GA, *n*	4,967	4,665		
Delivery room outcomes				
DCC duration seconds, median (IQR)	180 (180, 180)	180 (150, 180)	−8.0 (−9.8, −6.1)	<0.001
DCC ≥60 s, *n* (%)^a^	4,619 (96.3)	4,333 (95.1)	−1.2 (−2.1, −0.4)	0.003
DCC ≥180 s, *n* (%)^a^	3,746 (78.1)	3,204 (70.3)	−7.8 (−9.6, −6.0)	<0.001
Apgar @ 1 min <4, *n* (%)	72 (1.5)	90 (1.9)	0.5 (0.0, 1.0)	0.07
Apgar @ 5 min <8, *n* (%)	119 (2.4)	125 (2.7)	0.3 (−0.3, 0.9)	0.4
DR intubation, *n* (%)	4 (0.1)	4 (0.1)	0.0 (−0.1, 0.1)	0.9
Chest compression, *n* (%)	7 (0.1)	0 (0.0)	n/a	n/a
Delivery room epinephrine, *n* (%)	1 (0.0)	0 (0.0)	n/a	n/a
Cord gas pH < 7, *n* (%)	38 (0.8)	51 (1.0)	0.3 (−0.1, 0.7)	0.1
Cord gas base deficit ≥16, *n* (%)	36 (0.7)	56 (1.2)	0.5 (0.1, 0.9)	0.02
Neonatal outcomes				
Any breastfeeding, *n* (%)	4,844 (97.5)	4,543 (97.4)	−0.1 (−0.8, 0.5)	0.7
Exclusive breastfeeding, *n* (%)	3,271 (65.9)	3,271 (70.1)	4.3 (2.4, 6.2)	<0.001
NICU admissions, *n* (%)	255 (5.1)	299 (6.4)	1.3 (0.3, 2.2)	0.009
NICU admission diagnosis				
Evaluation for HIE, *n* (%)	48 (1.0)	71 (1.5)	0.6 (0.1, 1.0)	0.02
Therapeutic hypothermia, *n* (%)	9 (0.2)	18 (0.4)	0.2 (0.0, 0.4)	0.06
Hypoglycemia, *n* (%)	16 (0.3)	18 (0.4)	0.1 (−0.2, 0.3)	0.6
Respiratory distress, *n* (%)	118 (2.4)	127 (2.7)	0.3 (−0.3, 1.0)	0.3
Evaluation for sepsis, *n* (%)	21 (0.4)	41 (0.9)	0.5 (0.1, 0.8)	0.006
Early onset sepsis, *n* (%)	0 (0.0)	1 (0.0)	n/a	n/a
NICU interventions				
Antibiotics, *n* (%)	36 (0.7)	80 (1.7)	1.0 (0.5, 1.4)	<0.001
Respiratory support, *n* (%)	107 (2.1)	121 (2.6)	0.4 (−0.2, 1.1)	0.2
Nasal CPAP, *n* (%)	59 (1.2)	82 (1.8)	0.6 (0.1, 1.1)	0.02
Nasal IMV, *n* (%)	6 (0.1)	8 (0.2)	0.1 (−0.1, 0.2)	0.5
Intubation, *n* (%)	13 (0.3)	15 (0.3)	0.1 (−0.2, 0.3)	0.6
Inhaled nitric oxide, *n* (%)	3 (0.1)	9 (0.2)	0.1 (0.0, 0.3)	0.07
Transfusion, *n* (%)	10 (0.2)	14 (0.3)	0.1 (−0.1, 0.3)	0.3

^a^ A total of 171 patients from Pre-COVID-19 and 107 patients from COVID-19 period were excluded from difference analysis due to missing data.

### Neonatal outcomes

There was a significant increase in the exclusive breastfeeding rate (65.9% vs. 70.1%) and NICU admissions (5.1% vs. 6.4%) during the COVID-19 pandemic period. The frequency of NICU admissions, admission diagnosis, and NICU interventions are shown in Table 2. There was a significant increase in infants admitted to NICU for evaluation of hypoxic ischemic encephalopathy (HIE) (1% vs. 1.5%) and evaluation for sepsis (0.4% vs.0.9%) during the pandemic. There was an increase in the use of antibiotics (0.7% vs. 1.7%) and nasal CPAP (1.2% vs. 1.8%) during the COVID-19 pandemic period. The increases in overall NICU admissions and nasal CPAP were not significant after adjusting for baseline differences in GA, maternal diabetes, and maternal hypertension, however NICU admission for evaluation of HIE, evaluate for sepsis, and antibiotic use remained significant even after adjusting for the baseline differences (Table 3). Maternal hypertension was independently associated with an increase in the risk of NICU admission, NICU antibiotic, CPAP, and a decrease in the exclusive breastfeeding rate.

**Table 3:** Multivariate regressions analysis.

Outcome	Predictors	Odds ratio	95% CI	*p*-value
Exclusive breastfeeding				
	Study period	1.33	1.22–1.46	<0.001
	Gestational age, days	1.28	1.24–1.33	<0.001
	Maternal diabetes	0.75	0.68–0.83	<0.001
	Maternal hypertension	0.62	0.56–0.69	<0.001
NICU admission				
	Study period	1.16	0.97–1.39	0.09
	Gestational age, days	0.68	0.63–0.73	<0.001
	Maternal diabetes	1.10	0.90–1.34	0.4
	Maternal hypertension	1.80	1.49–2.18	<0.001
Evaluation for HIE				
	Study period	1.51	1.05–2.17	0.03
	Gestational age, days	0.95	0.82–1.10	0.5
	Maternal diabetes	1.13	0.75–1.68	0.6
	Maternal hypertension	2.28	1.57–3.32	<0.001
Cord gas base deficit ≥16				
	Study period	1.58	1.04–2.40	0.03
	Gestational age, days	1.00	0.84–1.19	1.0
	Maternal diabetes	1.02	0.62–1.66	0.9
	Maternal hypertension	3.14	2.04–4.83	<0.001
Evaluation for sepsis				
	Study period	2.07	1.21–3.52	0.008
	Gestational age, days	1.05	0.80–1.38	0.7
	Maternal diabetes	0.77	0.41–1.43	0.4
	Maternal hypertension	2.01	1.17–3.47	0.01
NICU antibiotic use				
	Study period	2.25	1.52–3.35	<0.001
	Gestational age, days	0.72	0.61–0.85	<0.001
	Maternal diabetes	0.74	0.47–1.16	0.2
	Maternal hypertension	1.73	1.16–2.57	0.007
NICU CPAP				
	Study period	1.36	0.96–1.91	0.08
	Gestational age, days	0.62	0.53–0.72	<0.001
	Maternal diabetes	1.15	0.79–1.67	0.5
	Maternal hypertension	1.59	1.09–2.33	0.02

## Discussion

In this single-center study, during the COVID-19 pandemic period, we observed an increase in maternal morbidities like hypertension and diabetes, severe metabolic acidosis in cord blood, exclusive breastfeeding, NICU admissions, and use of antibiotics, and respiratory support in infants born at ≥35 weeks GA and a decrease in three minutes DCC.

### Maternal morbidities

In our study, we observed a significant increase in maternal hypertension and diabetes during the pandemic. The baseline rate of maternal hypertension in the United States in 2019 was 16%, up from 13% in 2017 (8). In our study, the baseline maternal hypertension was already at 22%, much higher than that reported nationally. Many systematic reviews and meta-analyses have shown an increased risk of pre-eclampsia in pregnant mothers who had SARS-CoV-2 infection (9–11), with the risk being higher in those with symptomatic infection compared to asymptomatic and with severe infections (12, 13). The rate of SARS-CoV-2 infection in our study was relatively low, and a majority of those were identified due to asymptomatic screening. Despite the low SARS-CoV-2 infection rate, we had a significant increase in the rate of hypertension and diabetes in our study population.

The increase in maternal hypertension and diabetes in our study may be attributable to multiple factors such as decreased physical activity due to the shelter in place and remote work, significant impact on the socio-economic factors leading to stress, and increasing disparities for the marginalized population, including minorities. There was a significant decrease in access to care for all patients, including prenatal care worldwide. Even though telehealth was widely implemented, reports have shown decreases in overall care. The increase in maternal morbidities has been reported both nationally and internationally. There has been an increase in maternal hypertension and diabetes reported in North American epicenters of the pandemic (14–16). A Chinese study showed that pregnant women who experienced lockdown had an increase in HbA1C in those with gestational diabetes and an increase in pregnancy-induced hypertension in normoglycemic women (17). Italian studies also have shown an increase in gestational diabetes during the pandemic (18, 19). An Israeli study showed an increase in both hypertension and gestational diabetes during the pandemic (20). Another study from France showed that the glycemic control in GDM was poor during the pandemic (21). Other studies and a meta-analysis have shown a similar increase in gestational diabetes during the pandemic (22), especially in the Hispanic population (23). However, studies from France and Australia have not shown an increase in maternal diabetes or hypertension during or after the pandemic lockdown (24, 25).

### Delivery room outcomes

The increase in maternal morbidities has a negative effect on newborns. Maternal hypertension and diabetes both affect fetal well-being during labor. In our study, there was an increase in metabolic acidosis in the cord blood during the pandemic, and maternal hypertension was associated with three times the odds of severe metabolic acidosis. Other studies that have reported on cord gas values during the pandemic have not shown any difference (24, 26). One major difference between our study and the multicenter study from France (24) is the prevalence of maternal hypertension: their baseline hypertension was 5.4% compared to 22% in our study. Moreover, in contrast to a 4% increase in maternal hypertension in our study, there was no increase in their study.

In our center, the goal is to wait three minutes before clamping the cord if the infant is breathing by one minute of life and there is no concern for maternal bleeding. The decrease in the proportion of infants receiving three minutes of DCC is indirect evidence of suboptimal fetal/neonatal transition or a decrease in the well-being of the mother at the time of birth.

### NICU admissions

NICU admissions increased in the ≥35weeks GA population in our study during the pandemic. A systematic review of 38 studies evaluating the impact of the mitigation efforts against COVID-19 showed that there was a decrease in NICU admissions (27). Other studies have shown no change in NICU admissions (24, 28) and specifically a decrease in NICU admissions (29–31) for term infants during the COVID-19 pandemic. Differences in patient demographics may explain the differences between our study and others: we have a higher maternal hypertension rate (22%–26%), number of Hispanics (68%–73%), and majority of our patients are on public insurance. The negative effects of the pandemic were higher in the disadvantaged population, which adds to the worsening of the maternal and neonatal health outcomes in this population. An observational cohort study from UK neonatal research network showed a decrease in overall NICU admissions during the pandemic compared to the same time during the previous seven years. However, they did show an increase in transfer to higher level NICU in term infants during the pandemic (30).

### Exclusive breastfeeding

Encouragingly, we observed an increase in the exclusive breastfeeding rate during the study period. Similarly, observational studies from the UK research network and Spain showed an increase in the breastfeeding rate at discharge in term infants during the pandemic (30, 32). Many other studies have shown a decrease or plateauing in breastfeeding rate during the pandemic (28, 33–38). One study showed that there was no difference in breast milk feeding in those on public insurance compared to an increase in breastfeeding rates in those with private insurance (39). Despite the majority of our patient population being on public insurance, we showed an increase in the breastfeeding rate. The pandemic has had direct and indirect consequences on breastfeeding. During the lockdown, many face-to-face professional and peer supports were reduced. A survey from 2021 showed 42% of mothers felt breastfeeding was protected due to the lockdown, however, 27% of mothers struggled to get support and numerous barriers stemming from the lockdown, resulting in earlier cessation of breastfeeding (40). A published narrative review in 2021 included 12 studies looking at breastfeeding plans in women during the pandemic (41). Mothers reported positive breastfeeding experiences when they perceived more time for motherhood and negative breastfeeding experiences when mothers were separated from their newborns and had decreased family and professional support. In a recent study, Gribble et al. evaluated the guidelines around breastfeeding in mothers with SARS-CoV-2 infection from 101 countries (42). Despite WHO's strong recommendation early in the pandemic supporting skin-to-skin, breastfeeding, and rooming in with mothers with COVID-19, less than a quarter of the guidelines recommended them. In our institution, mothers who were positive for SARS-CoV-2 were counseled extensively on the benefits of breastfeeding and proper respiratory hygiene while breastfeeding and were given the option of having their infant room-in with them. Every one of them chose to room-in with their infant. The continued hands-on lactation support for all mothers during their hospital stay allowed for successful breastfeeding in all newborns, including those who were born to mothers positive for SARS-CoV-2 infection at the time of birth. Some of the visitation restrictions in the post-partum unit may have allowed the staff to spend more time with mothers supporting breastfeeding and uninterrupted time for the mother to breastfeed the newborn.

One limitation of our study is that it is from a single center. However, our findings should be generalizable to other public safety net hospitals that serve similar patient populations.

## Conclusion

During the COVID-19 pandemic period, we observed a significant increase in maternal morbidities and NICU admissions in infants born at ≥35 weeks gestation. The increase in NICU admissions during the COVID-19 pandemic was explained by maternal hypertension, but other adverse neonatal outcomes were only partly explained by an increase in maternal hypertension. Other socio-economic factors and social determinants of health need to be further explored to understand the full impact of the COVID-19 pandemic on neonatal outcomes. We also observed an increase in the exclusive breastfeeding rate during the pandemic, which is encouraging and likely attributable to uninterrupted lactation services in the postpartum unit to support breastfeeding.

## Data Availability

The raw data supporting the conclusions of this article will be made available by the authors, without undue reservation.
